# E-cadherin expression and bromodeoxyuridine incorporation during development of ovarian inclusion cysts in age-matched breeder and incessantly ovulated CD-1 mice

**DOI:** 10.1186/1477-7827-5-14

**Published:** 2007-04-11

**Authors:** Jean S Fleming, H James McQuillan, Melanie J Millier, Clare R Beaugié, Vicki Livingstone

**Affiliations:** 1 Eskitis Institute of Cell & Molecular Therapies, Griffith University Nathan campus, Nathan, QLD 4111, Australia; 2 Department of Anatomy and Structural Biology, University of Otago School of Medical Sciences, PO Box 913, Dunedin, New Zealand; 3 Department of Preventive & Social Medicine, University of Otago Health Sciences, PO Box 913, Dunedin, New Zealand

## Abstract

**Background:**

Female CD-1/Swiss Webster mice subjected to incessant ovulation for 8 months and 12-month breeder mice both developed ovarian inclusion cysts similar to serous cystadenomas. The majority of cysts appeared to be dilated rete ovarii tubules, but high ovulation number resulted in more cortical inclusion cysts. We hypothesized that comparison of inclusion cyst pathology in animals of the same age, but with differences in total lifetime ovulation number, might allow us to determine distinguishing characteristics of the two types of cyst.

**Methods:**

Ovaries from breeder mice (BR) or females subjected to incessant ovulation (IO) were compared at 6-, 9- and 12-months of age. Ovaries were serially sectioned and cysts characterized with regard to location and histology, E-cadherin immunoreactivity and rates of BrdU incorporation.

**Results:**

Inclusion cysts developed with age in BR and IO ovaries. The majority of cysts were connected to the ovarian hilus. Two cortical inclusion cysts were observed in ten IO ovaries and one in ten BR ovaries. Low or no E-cadherin immuno-staining was seen in the OSE of all mice studied. Conversely, strong membrane immuno-staining was observed in rete ovarii epithelial cells. Variable E-cadherin immunoreactivity was seen in cells of hilar inclusion cysts, with strong staining observed in cuboidal ciliated cells and little or no staining in flat epithelial cells. Two of the three cortical cysts contained papillae, which showed E-cadherin immuno-staining at the edge of cells. However hilar and cortical cysts were not distinguishable by morphology, cell type or E-cadherin immunoreactivity. BrdU incorporation in cyst cells (1.4% [95% CI: 1.0 to 2.1]) was greater than in OSE (0.7% [95% CI: 0.4 to 1.2]) and very few BrdU-labeled cells were observed in rete ovarii at any age. Incessant ovulation significantly increased BrdU incorporation in OSE of older animals.

**Conclusion:**

These experiments confirm ovarian inclusion cysts develop with age in the CD-1 mouse strain, irrespective of total ovulation burden. We conclude longer periods of incessant ovulation do not lead to significant changes in inclusion cyst formation or steroidogenesis in CD-1 mice and inclusion cyst type can not be distinguished by morphology, cell proliferation rate or E-cadherin immunoreactivity.

## Background

Our previous studies demonstrated that ovaries of CD-1/Swiss Webster mice subjected to incessant ovulation (IO) had changes suggestive of a precancerous state, such as an increased number of surface invaginations, stratification of the ovarian surface epithelium (OSE) and inclusion cyst formation [1,2]. Dilation of the rete ovarii tubules to form serous inclusion cysts also occurred with age in breeding females with a much lower total ovulation number [2]. However these studies compared ovaries from incessantly ovulated ovaries and breeders of different ages. We hypothesized that comparison of inclusion cyst pathology in animals of the same age, but with large differences in total lifetime ovulation number, might allow us to distinguish better between cortical and rete ovarii cysts. We have therefore compared age-matched mice subjected to incessant ovulation or repeated pregnancy for up to 12 months and assessed ovarian histology, rates of cell division and expression of the epithelial marker E-cadherin.

E-cadherin is a regulator of the differentiated epithelial cell phenotype, with key roles in the formation of cell adherens junctions and the establishment of epithelial polarity [3]. Mutation or abnormal expression of E-cadherin is known to be carcinogenic in a variety of epithelial tissues [4-6]. The OSE is continuous with the mesothelium covering the peritoneal cavity and is therefore better classified as a mesothelium, rather than a true epithelium [7]. Normal OSE and peritoneal mesothelial cells express little to no E-cadherin [8,9], but transformed OSE cells and cells from primary epithelial ovarian cancer (EOC) have increased E-cadherin expression [9-12]. Expression of exogenous E-cadherin in OSE cells can induce a mesenchymal-to-epithelial transition in the OSE, resulting in the expression of cytokeratins and a cobblestone epithelial appearance in culture [11]. Loss of E-cadherin expression predicts poor prognosis, especially in late-stage, serous EOC [3]. E-cadherin protein has been reported to be absent from the cells of the human rete ovarii [13], although it appears to be present in the mesonephros and mesonephric tubules of the fetal mouse ovary [14], from which the adult rete ovarii are derived. We therefore measured E-cadherin expression in our model of incessant ovulation, to compare expression in the cells lining inclusion cysts with that in the OSE or rete ovarii.

## Methods

### Animals

Experiments were approved by the University of Otago Animal Ethics Committee. The dissection, fixation, and processing of the ovaries has been described previously, as has estimation of total lifetime ovulation number [1,2]. Female out-bred CD-1/Swiss Webster mice were housed in two groups in a temperature- and light-controlled facility; (1) in breeding pairs (BR group) with one female housed with a male Swiss Webster mouse and (2) in a cage divided by a perforated screen, with two females housed beside, but not in contact with a single male mouse, to induce incessant ovulation (IO group) and prevent breeding [1]. Breeder females were separated from their males at the appropriate age, on weaning of the last litter. Mice were housed in these environments from age of weaning until 6, 9 or 12-months of age. Blood samples were obtained on euthanasia from all mice and cyst fluid was aspirated on ovarian dissection where possible.

### Determination of stage of estrous cycle

Vaginal cytology was monitored in BR and IO mice using the lavage method [15] to check the mice were cycling regularly. Vaginal smears were taken in the morning before 1000 hours for no more than five consecutive days. Once they had reached the appropriate age, vaginal smears were taken to determine stage of cycle and the animals were killed at between 1300 and 1500 hours on the afternoon of the 0600 hours estrus smear.

### Plasma collection

Mice were anesthetized with halothane gas and cardiac puncture exsanguination was performed using a 26 gauge needle and a heparinized syringe. One to 2 ml of blood was obtained from each animal and the plasma was stored at -80°C until assay.

### Cyst fluid collection

Fluid was aspirated from ovaries containing visible cysts, using an ultra-fine insulin needle and 1.0 ml syringe. Fluid was frozen at -80°C before use and diluted 1/20 before estradiol-17β radioimmunoassay. Between 20 and 950 μl cyst fluid was obtained from nine ovaries. Cyst fluid was assayed for estradiol-17β only.

### Determination of inclusion cysts and dilated rete ovarii

One ovary from 10 mice from the IO and BR treatment groups at each age was removed at time of death and fixed in 4% paraformaldehyde for 18 hours, before embedding in paraffin and serial sectioning at a 4 μm thickness. Every 25^th ^section was stained with hematoxylin and eosin (H&E) and the number of cystic structures and/or dilated rete ovarii determined [2].

### BrdU incorporation

Mice were injected three times i.p. with 3 mg/100 g body weight of BrdU, dissolved in sterile isotonic saline, at 0700 h, 0900 h and 1100 h, before euthanasia at 1300 h. The harvested ovaries were fixed in 4% paraformaldehyde, embedded in paraffin and BrdU immunohistochemistry was performed on 4 μm sections adjacent to the H&E stained sections containing cysts or rete ovarii from each animal. Antigenic sites were exposed by incubation with 5 μg/ml proteinase K (Sigma-Aldrich Inc., St Louis, MO USA)for 25 min at 37°C and DNA denatured by incubation in 4 M HCl for 10 min at room temperature. The acid was neutralized by washing in 0.1 M sodium borate for 10 min at room temperature and endogenous peroxidase quenched by incubation with 0.3 % (v/v) H_2_O_2 _in methanol. Sections were incubated with 50 μl/section 1:50 dilution of a monoclonal mouse anti-BrdU antibody (Dako, Carpentaria, USA) in phosphate buffered saline containing 1% bovine serum albumin (PBS-BSA; Sigma-Aldrich) overnight at 4°C. Negative control slides were incubated in PBS-BSA without primary antibody. Sections were incubated with a 1:200 dilution of biotin-conjugated goat anti-mouse IgG (Amersham GE Healthcare UK Ltd, Buckinghamshire HP7 9NA, England), before incubation with streptavidin biotinylated horseradish peroxidase (Amersham) for one hour at room temperature. Labeled cells were visualized with diaminobenzidine (DAB; Vector Laboratories Inc., Peterborough, UK). All OSE, rete ovarii and cyst epithelial cells in each stained section were scored for nuclear BrdU staining and % incorporation calculated as the number of stained cells as a percentage of the total number of cells counted for each cell type. All ovaries were collected on the afternoon of estrus and thus all ovaries contained ovulation sites, but not all sections analyzed for BrdU incorporation contained obvious ovulation sites.

### E-cadherin immunohistochemistry

E-cadherin immunohistochemistry was performed after antigen retrieval by microwaving in boiling 1 mM EDTA, pH 8.0, using a rat anti-mouse E-cadherin monoclonal primary antibody (Clone ECCD-2, Isotype Rat IgG_2a_, Zymed Laboratories, San Francisco, CA, USA; 100 μg/ml), incubated overnight at 4°C. Sections were then incubated with a goat anti-rat biotinylated secondary antibody (1:100 dilution; Zymed), before incubation with streptavidin biotinylated horseradish peroxidase (1:50 dilution; Amersham) for one hour at room temperature. Labeled cells were visualized with DAB and sections counterstained with 50% Gill's hematoxylin.

### E-cadherin immunoblotting

Samples of mouse whole ovary, uterus, small intestine (positive controls) and skeletal muscle (negative control) were snap frozen, pulverized in liquid nitrogen and the protein extracted in SDS reducing buffer as previously described [16]. Protein concentrations were determined using a bicinchoninic acid protein assay kit (Pierce Biotechnology, Rockford, IL). Protein extracts were separated on discontinuous 8% SDS-PAGE gels and electroblotted onto polyvinylidene difluoride membranes (PVDF, Roche Pharmaceuticals, Auckland, New Zealand). A 10–250 kDa pre-stained protein marker was loaded onto all gels. Blots were probed for 1 h at room temperature with the E-cadherin primary antibody (Zymed Clone ECCD-2; 1 μg/ml) in phosphate buffered saline, pH 7.4, containing 0.1% (v/v) Tween 20 and 3% (w/v) skim milk powder (PBS-TM). Membranes were washed and incubated with horseradish-peroxidase-conjugated goat anti-rat immunoglobulins (Zymed; diluted 1:2000 in PBS-TM) for 1 h at room temperature, followed by incubation with streptavidin biotinylated horseradish peroxidase (Amersham) at a dilution of 1:5000 in TBS for 1 hour, prior to detection of bound antibody with the ECL chemiluminescent detection system (Amersham) according to the manufacturer's directions [17].

### Plasma steroid radioimmunoassay

Radioimmunoassay (RIA) kits were obtained from Diagnostic Systems Laboratories (DSL, Webster, Texas, USA) to measure the concentration of estradiol-17β (DSL-39100 3^rd ^generation 17β-Estradiol RIA Kit), total testosterone (DSL-4000 ACTIVE^® ^Testosterone Coated-Tube Radioimmunoassay Kit), and androstenedione (DSL-3800 ACTIVE^® ^Androstenedione Coated Tube Radioimmunoassay Kit). Each RIA was performed according to the manufacturer's instructions and all plasma samples were assayed in duplicate. Five separate assays containing samples from a random selection of treatment groups were carried out. Plasma samples were diluted in assay buffer containing 0.1% (w/v) bovine serum albumin (BSA). Dilution factors ranged between 1 and 10, depending on the amount of plasma available for each sample. Three independent assays were carried out for testosterone and androstenedione measurements, none of which required sample dilution. RIA data were analyzed using a universal assay calculator computer software package (BIOSOFT AssayZap V 2.51, 1995). The sample intra-assay coefficient of variation (CV) ranged from 8.6% to 12.9% for the estradiol-17β assays, 17.8% to 18.6% for testosterone and 7.5% to 16.2% for the androstenedione assays. Inter-assay CVs were determined using results for high and low quality control (QC) samples across all assays. Inter-assay CV for estradiol-17β: low QC (10 pg/ml) = 17.0%, high QC (30 pg/ml) = 6.5%; testosterone: low QC (0.5 ng/ml) = 19.7%, high QC (5.0 ng/ml) = 5.2%; androstenedione: low QC (0.9 ng/ml) = 13.3%, high QC (6.0 ng/ml) = 15.0%.

### Data Analysis

All statistical analyses were conducted using SPSS 14.0 (SPSS, Chicago, USA) or SAS 9.1.2 (SAS Institute Inc., Cary, NC, USA). A P-value < 0.05 was considered to be statistically significant.

### BrdU incorporations analysis

Group mean BrdU incorporations were compared using negative binomial regression. Age group, treatment group and the interaction of age and treatment groups were included as explanatory variables in the model. Pairwise comparisons with a Bonferroni correction were performed to determine which groups were significantly different if differences were found in the regression analysis. Analyses were performed for OSE and cyst cells separately. Negative binomial regression using generalized estimating equations was used to compare the OSE BrdU incorporation rate with the cyst cell incorporation rate in those animals that had cysts. The generalized estimating equation accounts for correlation among observations from the same animal.

### Steroid data analysis

For all analyses, testosterone and androstenedione plasma concentrations were log transformed before analysis, because of their positively skewed distributions. The original data for estradiol were used in the analysis. Group mean values in steroid data were compared using a 2-way ANOVA grouped on age (6, 9 12-months) and treatment (BR, IO). The main effects and the interaction were included in the model. Pairwise comparisons with a Bonferroni correction were performed to determine which groups were significantly different, if differences were found with the 2-way ANOVA. A linear mixed model was used to compare the mean estradiol in plasma with the mean estradiol in cyst fluid for animals that had a cyst. The fixed effect was section (plasma, cyst) and the random effect was the animal. The random animal effect allows us to model possible correlations between measurements from the same animal. Plasma steroid levels were initially compared in animals with ovarian inclusion cysts or dilated rete ovarii and those without using an independent samples t-test. To adjust for the potential confounding effect of age group, a linear regression analysis was also performed with cyst presence and age group as explanatory variables in the model.

## Results

### Estimated ovulation numbers

Total lifetime ovulation number was estimated from the records of the breeding pairs, as described previously [1,2]. Although litter size declined only minimally between 6 and 12-months of age, a decrease in litter frequency led to a significant decline in the number of pups born per month to the breeding pairs. The mean estimated total ovulation number for each treatment group and age is shown in Table 1. These values are estimated from average litter size over lifetime, as well as minimum and maximum age of puberty and estrous cycle length and are therefore under-estimations of true ovulation burden [1]. Body weight increased with age in IO mice, but not from 6 months of age in BR mice (Table 1).

**Table 1:** Numbers (n) of mice, age in days (sd), mean body weight in grams (sd) and estimated mean total lifetime ovulation number (OV#: minimum – maximum estimate) in mice subjected to incessant ovulation (IO) or repeated pregnancy (BR) from 6–12 months (m) of age.

**Group**	**Treatment**	**Age (days)**	**n**	**Body weight (g)**	**Estimated OV# (min – max)**
6 m	IO	193 (3)	10	43 (3)	336 (284–388)
	BR	190 (6)	17	45 (3)	161 (129–193)
9 m	IO	275 (12)	14	50 (2)	642 (585–698)
	BR	300 (3)	19	42 (3)	188 (155–222)
12 m	IO	374 (1)	18	53 (2)	720 (561–878)
	BR	392 (9)	16	45 (4)	286 (206–367)

OV# was estimated as described previously [1].

### Frequency of ovarian inclusion cysts and dilated rete ovarii

**Figure 1:**
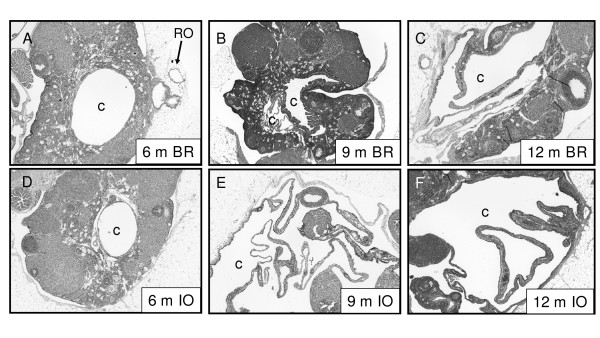
Cyst size and cellular metaplasia increased with age in both BR (A-C) and IO (D-F) mice. **A**. Intraovarian cyst (c) in an ovary from a 6-month BR mouse. Serial sectioning of this ovary showed this cyst originated in the hilus of the ovary. Dilated rete ovarii tubules (RO) are present in the extra-ovarian fat. **B**. Ovary from a 9-month BR mouse, containing an intraovarian cortical cyst with many papillae (right) and a hilar cyst (lower left). **C**. Ovary from a 12-month BR mouse containing a large, collapsed hilar cyst, from which cyst fluid has been aspirated. **D**. Cyst in the medulla of a 6-month IO mouse, which was shown to be connected to the hilus of the ovary on serial sectioning. **E**. Collapsed cyst of unknown origin in the ovary of a 9-month IO mouse. The original diameter of this ovary was 11 mm before aspiration of 950 μl of cyst fluid. **F**. Hilar cyst with extensive papillae formation in an ovary from a 12-month IO mouse.

Ovarian cysts were common across all age and ovulation groups on inspection of histological sections (Table 2). Cyst size and cellular metaplasia increased with age (Figure 1). Serial ovarian sectioning showed the majority of cysts were hilar in origin and appeared to be dilated rete ovarii, but in one case the cyst was so large its origin could not be determined. True cortical inclusion cysts, with no connection to the ovarian hilus, were rare in both groups. Of the four cortical inclusion cysts observed on serial sectioning, three were in 6-, 9- and 12-month IO ovaries and one was in a 9-month old BR ovary (Figure 1 and Table 2). This breeder had had eight litters and a total of 114 pups in her lifetime, with an estimated ovulation number of 182–250, similar to the other animals in her group.

**Table 2:** Numbers of cysts in ovaries of mice subjected to incessant ovulation (IO) or repeated pregnancy (BR) from weaning until 6–12 months of age. Ten ovaries from each group were serially sectioned to determine the extent and origin of the cysts and rete ovarii tubules (RO). The number of ovaries containing cysts of different types is given. Ovaries could contain more than one type of cyst. In one case the cyst was so large that, after aspiration of cyst fluid on dissection, the origin of the cyst could not be determined. There were no differences in the frequency of cortical cysts between groups.

**Age**	**6-month**		**9-month**		**12-month**	
**Group**	**IO**	**BR**	**IO**	**BR**	**IO**	**BR**

Number of ovaries examined	10	10	10	10	10	10
Ovaries containing cysts	6	6	8	8	7	8
Ovaries with extra-ovarian cysts	1	2	2	2	0	5
Ovaries with hilar inclusion cysts	5	5	6	6	6	5
Ovaries with cortical inclusion cysts	0	0	1	1	1	0
Ovaries with cysts of unknown origin	0	0	1	0	0	0

### Cyst cell morphology

Most cysts were typically lined with a single layer of epithelial cells, ranging in shape from flat to cuboidal or columnar, with cilia frequently present on the cuboidal and columnar cells. Two of the three cortical inclusion cysts were lined only with cuboidal and columnar cells, with a noticeable absence of any flat cells. Many of the columnar cells had nuclei with an apical location and no cilia were seen on these cells. Papillae were observed in both dilated rete ovarii and cortical inclusion cysts with no apparent connection to the hilus. Cortical cysts were distinguished from cystic follicles by lack of a thecal layer beneath the cyst epithelium and by the presence of flattened or ciliated cells.

### E-cadherin immunoblotting and immunohistochemistry

**Figure 2:**
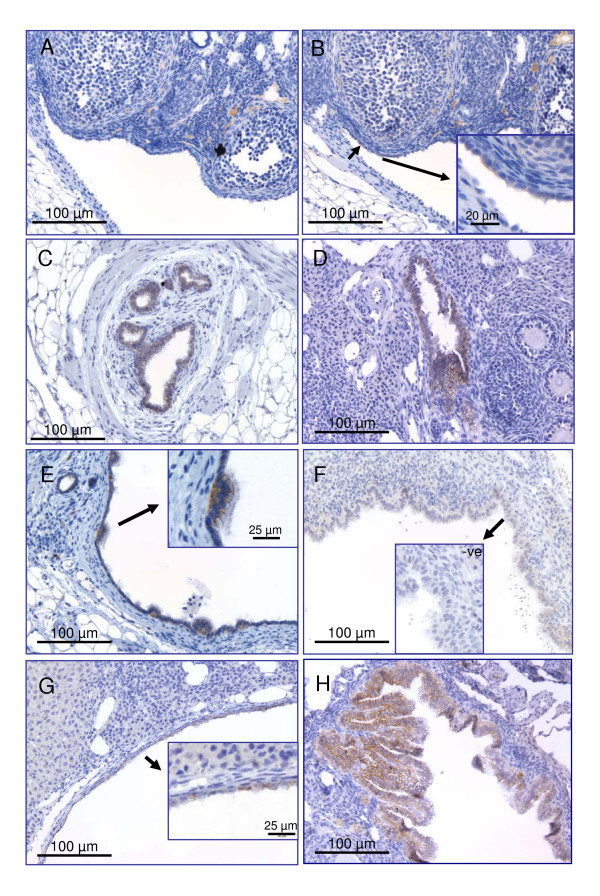
E-cadherin immunoreactivity in the epithelial cells of cystic mouse ovaries. **A**. OSE to mesothelial junction of an ovary from a 6-month BR mouse; negative control without primary antibody. **B**. A small amount of E-cadherin immunoreactivity can be seen in the OSE, but not the mesothelium of a serial section to A. **C**. Strong junctional E-cadherin immuno-staining in extra-ovarian rete ovarii from a 6-month IO mouse. **D**. E-cadherin immuno-staining in an intra-ovarian rete ovarii tubule from a 6-month IO mouse. **E**. Ciliated cells lining a hilar ovarian inclusion cyst show E-cadherin immunoreactivity, whereas flatter cells lining the cyst are unstained. **F**. Cells lining a cortical inclusion cyst from a 9-month IO ovary showing very little membrane-bound E-cadherin immuno-staining. Insert shows the negative control without primary antibody. **G**. Lack of E-cadherin junctional immuno-staining in some, but not all cells lining an inclusion cyst connected to the ovarian hilus, in a 6-month IO ovary. **H**. Strong membrane-limited E-cadherin immunoreactivity in cells of papillae in a cortical inclusion cyst from a 12-month IO ovary.

No or extremely low levels of E-cadherin immunoreactivity were observed in the ovaries of BR or IO ovaries at any age, using the immunohistochemical conditions employed in this study. In particular, the OSE was essentially negative for E-cadherin immunostaining. Invaginations of the OSE did not show increased E-cadherin immunoreactivity (data not shown), but some staining was observed occasionally in the OSE close to the OSE-mesothelial junction (Figure 2A and 2B). In contrast, strong immunoreactivity was observed on the edges of both intra- and extra-ovarian rete ovarii tubule cells (Figure 2C and 2D). In large hilar cysts, E-cadherin immunoreactivity was more variable and flat cells had no membrane-limited staining, whereas staining around ciliated and cuboidal cells remained strong (Figure 2E and 2G). One cortical inclusion cyst had little E-cadherin immunoreactivity (Figure 2F), whereas the two with extensive papillae formation showed strong membrane staining (Figure 2H).

**Figure 3:**
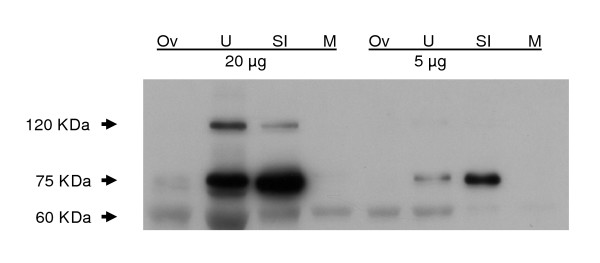
E-cadherin immunoblot of 5 μg and 20 μg extracted protein from whole mouse ovary (Ov), uterus (U), small intestine (SI) and skeletal muscle (M).

Immunoreactive bands of around 120 kDa and 75 KDa [10,18] were seen on immunoblotting of 5 or 20 μg protein extracted from uterus or small intestine, but no or very low signals were observed in extracts of whole ovary or skeletal muscle. An immunoreactive band was also seen at 60 KDa, particularly in uterine and ovarian extracts (Figure 3). Non-specific immunoreactivity migrated at the gel front in all lanes with 20 μg protein extract (data not shown).

### BrdU incorporation

**Figure 4:**
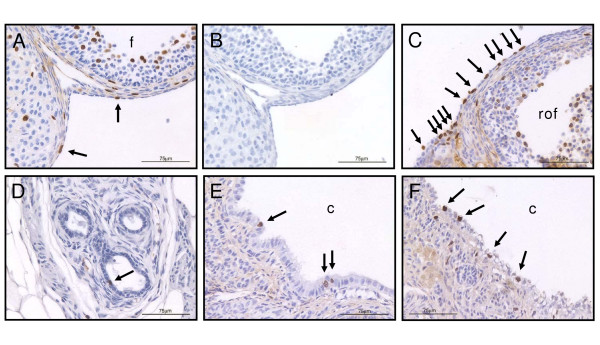
Representative examples of anti-BrdU immunohistochemistry in the ovaries of IO and BR mice. **A**. BrdU immuno-positive cells in the OSE (arrows) adjacent to a follicle (foll) and a corpus luteum in a 9-month IO ovary on the afternoon of estrous. Note the labeled cells in the theca and stroma just beneath the OSE. **B**. Negative control (no primary antibody added) of an adjacent section to A. **C**. Numerous BrdU immuno-positive nuclei (arrows) in the OSE at the base of a recently ovulated follicle (rof) in a 6-month BR ovary. **D**. A single BrdU immuno-positive cell (arrow) is present in this group of three rete ovarii tubules from a 9-month BR ovary. **E**. BrdU incorporation in cells present in a hilar cyst (c) in a 12-month BR ovary. **F**. BrdU incorporation in cells, including hobnail cells with clear cytoplasm, lining a cortical cyst in a 12-month IO ovary. Bars = 75 μm; original magnification 200 ×.

BrdU immunohistochemically-stained nuclei were observed in sections of all ovaries, regardless of age or ovulation number. Within the ovaries, numerous granulosa and theca cells in the developing follicles showed BrdU incorporation, with stained nuclei also evident in the corpora lutea and stroma (Figure 4).

The percentages of BrdU-stained nuclei in OSE, cyst epithelia or rete ovarii tubules are shown in Table 3. Numbers of BrdU-stained nuclei in ovarian epithelial cells were low and the highest rates of incorporation were observed in the cyst epithelium, at levels of around 1–2% (Table 3). BrdU incorporation was evident in < 1% of OSE nuclei, although numbers varied greatly between animals of the same age and stage of estrous cycle. Figure 4 shows representative examples of BrdU immunohistochemistry in the OSE, rete ovarii and epithelial cells lining cysts. OSE cell labeling was most frequently seen at the base of fresh ovulation sites. Labeled cuboidal cells also appeared at random throughout the OSE. No increase in BrdU incorporation was noted in the region of the transition between the OSE and the peritoneal mesothelium, at the hilus of the ovary (data not shown).

**Table 3:** Median (IQR) percentages of BrdU-stained nuclei observed in the OSE and cyst epithelia and median (range) of BrdU-stained nuclei observed in normal rete ovarii of mice of different ages and total lifetime ovulation number.

**Group**	**Treatment**	**OSE n = 59**	**Cyst n = 36**	**Rete ovarii n = 16 Median(range)**
6 m	IO	1.3 (0.0, 2.6)	2.2 (0.0, 2.8)	0.0 (0.0, 2.0)
	BR	0.4 (0.1, 3.3)	0.9 (0.6, 4.0)	0.0 (0.0, 0.0)
9 m	IO	0.7 (0.2, 1.3)	0.5 (0.1, 3.2)	0.0 (-) *
	BR	0.1 (0.0, 0.2)	1.5 (0.8, 2.3)	0.3 (0.0, 0.7)
12 m	IO	0.2 (0.0, 0.7)	0.0 (0.0, 1.9)	- **
	BR	0.1 (0.0, 0.1)	0.3 (0.0, 1.9)	0.0 (0.0, 2.0)

* Normal rete ovarii tubules were observed in only one animal in this group.

** No normal, undilated rete ovarii tubules were observed in ovaries from this group.

BrdU incorporation rate was examined in ovarian cyst epithelia from 36 animals. Data for each age and treatment group combination are described in Table 3. Neither the main effects of age (P = 0.500) nor treatment group (P = 0.761) nor the interaction term (P = 0.721) were statistically significant for cyst cells, suggesting BrdU incorporation did not decline significantly with age in cyst cells. BrdU incorporation in the OSE was compared with that in cyst epithelium of the same ovary in the 36 animals that had cysts. There was a statistically significant difference in BrdU incorporation rate in OSE compared with cyst cells (P = 0.011). The predicted percentage proliferation rate in OSE was 0.7% (95% CI: 0.4 to 1.2) and in cysts was 1.4% (1.0 to 2.1). The average BrdU incorporation rate for the OSE was 0.50 times that of the cysts (95% CI: 0.30 to 0.85).

Data on BrdU incorporation rate in the normal rete ovarii tubules were available for 16 animals. Stained nuclei were only observed in five (31%) of the rete ovarii samples observed. The median (IQR) percentage BrdU incorporation rate was 0.0 (0.0, 0.5). As the proliferation rates were so low, differences between age groups or treatment groups were not tested.

### Incessant ovulation increased BrdU incorporation in the OSE

**Figure 5:**
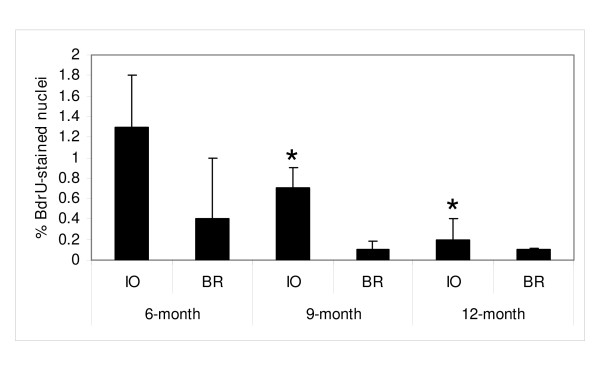
BrdU incorporation in the OSE of BR and IO mice, with age (mean +/- one s.d.). Numbers of BrdU-stained nuclei declined with age, but significantly higher numbers of stained nuclei were counted in the OSE from IO ovaries compared with BR ovaries (P = 0.002). All ovaries were collected on the afternoon of estrus.

Data on BrdU incorporation in OSE were available for 59 animals and are shown in Figure 5. BrdU incorporation declined with age in the OSE. The main effects of age and treatment were statistically significant (P < 0.001 and P = 0.002 respectively), but the interaction was not (P = 0.089). The BrdU incorporation rate in the IO group was 3.1 times (95% CI: 1.5 to 6.4) the BrdU incorporation in the BR group. The BrdU incorporation rate was significantly higher for the 6-month compared to the 9-month (incidence rate ratio = 3.7, 95% CI: 1.6 to 8.2, P = 0.002) and 12-month mice (incidence rate ratio = 7.9, 95% CI: 3.3 to 19.0, P < 0.001). There was a tendency towards BrdU incorporation being higher in the 9-month compared with the 12-month mice, but this was not statistically significant (incidence rate ratio = 2.2, 95% CI: 0.9 to 5.3, P = 0.098).

### Rates of apoptosis

Very few apoptotic epithelial cells were detected in OSE, cysts or rete ovarii using either active caspase-3 immunohistochemistry or TUNEL *in situ *hybridization (data not shown), despite numerous positive cells in atretic follicles. In the 100 ovarian sections analyzed, a total of five OSE cells and four cyst cells labeled positive for the active caspase-3 protein. These were observed in ovaries from all ages and treatment groups. In all ovarian sections analyzed, no rete ovarii cells labeled positive for the active caspase-3 protein and two positive cells were seen after TUNEL labeling.

### Plasma steroid concentrations across ages and ovulation groups

The plasma steroid concentrations for each age and ovulation group combination are described in Table 4. Estradiol-17β concentrations are described by the mean and standard deviation and those of testosterone and androstenedione are described by the median and inter-quartile range (IQR). Separate 2-way ANOVAs for each of the plasma steroid concentrations showed there was no interaction effect, i.e. the effect of treatment did not differ by age. The P-values for estradiol, testosterone and androstenedione were 0.462, 0.619 and 0.718, respectively. There was also no treatment effect (P = 0.984, 0.915 and 0.284). There was a significant decline with age in testosterone (P = 0.001) and androstenedione (P < 0.001) concentrations, but there were no significant differences in circulating estradiol-17β between age groups (P = 0.178). For testosterone, the geometric mean was significantly higher for the 6-month compared with the 9-month (P = 0.002) and 12-month mice (P = 0.003). Likewise for androstenedione, the geometric mean was significantly higher for the 6-month mice compared with 9-month (P = 0.009) and 12-month mice (P < 0.001).

**Table 4:** Mean (sd) plasma estradiol-17β concentration and median (IQR) plasma testosterone and androstenedione concentrations in incessantly ovulating (IO) and breeder (BR) mice killed on the afternoon of estrus, at 6-12 months (m) of age.

**Age**	**Treatment**	n*	**Estradiol-17β (pg/ml) mean (sd)**	**Testosterone (ng/ml) median (IQR)**	**Androstenedione (ng/ml) median (IQR)**
6 m	IO	19	17.82 (10.61)	0.37 (0.28, 0.64)	0.64 (0.47, 1.41)
	BR	15	22.05 (9.35)	0.50 (0.41, 0.80)	0.63 (0.52, 0.84)
9 m	IO	12	18.96 (12.79)	0.24 (0.18, 0.26)	0.48 (0.38, 0.67)
	BR	15	16.66 (9.58)	0.24 (0.12, 0.48)	0.40 (0.30, 0.53)
12 m	IO	16	24.49 (13.60)	0.27 (0.15, 041)	0.43 (0.29, 0.53)
	BR	14	22.71 (11.38)	0.19 (0.15, 0.32)	0.32 (0.30, 0.46)

* n = number of animals analyzed per group

### Does the level of estradiol-17β in cyst fluid differ from that in plasma from the same animal?

Gross examination of the ovaries on dissection revealed protruding fluid-filled cysts of >2 mm diameter in some older IO and BR animals. An adequate amount of cyst fluid was extracted for estradiol-17β RIA analysis in nine of these cysts and compared with plasma estradiol-17β levels in the same animal to investigate any possible differences in steroid concentrations between these two sources. Data on plasma estradiol was available for 7 of the 9 animals with cysts. The linear mixed model found no statistically significant differences in mean estradiol-17β levels between the cyst fluid and the plasma (P = 0.102). The mean plasma estradiol-17β in the nine animals where cyst fluid was obtained was 29.5 pg/ml (95% CI: 12.3 to 46.6), whereas the mean estradiol-17β in cyst fluid was 44.9 pg/ml (95% CI: 29.3 to 60.5). Although not statistically significant, there was a tendency for the estradiol concentration to be higher in the cyst fluid than in the plasma (difference in means = 15.42, 95% CI: [-3.85 to 34.69]), as might be expected in fluid sampled from closer to the site of estradiol synthesis.

### Does the presence of ovarian cysts or dilated rete ovarii affect plasma steroid levels?

#### Estradiol-17β

Plasma estradiol-17β concentrations were compared in animals with (n = 46) and without (n = 9) ovarian inclusion cysts or dilated rete ovarii. The mean (sd) for the animals with cysts was 17.4 (9.8) and for those without was 23.3 (11.4). There was not a statistically significant difference in estradiol between the animals with or without cysts based on the independent samples t-test (P = 0.124) or the regression analysis that adjusted for age (P = 0.129). The unadjusted difference in means for the group with cysts compared to the group without was -5.8 (95% CI: -13.1 to 1.6). The adjusted difference of means for the two groups was -5.7 (95% CI: -13.1 to 1.7). Therefore, although not statistically significant, there was a tendency for the animals with cysts to have lower estradiol than the animals without cysts.

#### Testosterone

Testosterone concentrations were compared in animals with (n = 40) and without (n = 10) ovarian inclusion cysts or dilated rete ovarii. The median (IQR) for the animals with cysts was 0.28 (0.17–0.47) and for those without was 0.46 (0.21–0.51). There was not a statistically significant difference in testosterone between the animals with or without cysts based on the independent samples t-test (P = 0.735) or the regression analysis that adjusted for age (P = 0.460). The unadjusted ratio of geometric means for the group with cysts compared to the group without was 0.92 (95% CI: 0.58–1.47). The adjusted ratio of geometric means for the group with cysts compared to the group without was 0.86 (95% CI: 0.57–1.29).

#### Androstenedione

Data on androstenedione were available for 47 animals with cysts and 11 animals without cysts. The median (IQR) for the animals with cysts was 0.46 (0.32 – 0.66) and for those without was 0.50 (0.31–0.69). There was not a statistically significant difference in androstenedione between the animals with or without cysts based on the independent samples t-test (P = 0.593) or the regression analysis that adjusted for age (P = 0.489). The unadjusted ratio of geometric means for the group with cysts compared to the group without was 0.91 (95% CI: 0.65–1.28). The adjusted ratio of geometric means for the group with cysts compared to the group without was 0.90 (95% CI: 0.67–1.22).

## Discussion

In these experiments, the presence of all ovarian cysts increased with age rather than total lifetime ovulation number, as has been previously reported for both mice [1,2] and women [7]. The majority of the cystic structures appeared to be dilated rete ovarii tubules. Cortical inclusion cysts, with no connection to the hilus of the ovary, were extremely rare, as in our previous observations in animals that were not age-matched [2]. The histology of the cortical inclusion cyst epithelia, including lack of a thecal cell layer, low rates of apoptosis, the presence of ciliated or flattened cells and E-cadherin immunoreactivity, suggested these cysts were not follicles that had failed to ovulate, although this possibility cannot be completely ruled out.

Although the observed cysts were large and the cyst epithelia showed some dysplasia, no enlarged or abnormal nuclei or overt epithelial neoplasia were observed. Dilation of the rete ovarii with age has been noted previously in CD-1 (Swiss Webster) mice [2,19] and appears to be a feature of this outbred strain, since similar cysts are not found in 12-month virgin or breeder inbred C57/BL6 or 9-month outbred Quackenbush mice (J. S. Fleming, unpublished observations). Recent studies by Clark-Knowles and colleagues showed conditional knockout of the *Brca1 *gene in the OSE of FVB mice, led to an increase in preneoplastic changes in ovarian biology, including OSE invagination, epithelial cell hyperplasia and small inclusion cysts, but did not give rise to ovarian cancers, up to 8 months after inactivation of *Brca1 *in the OSE [20]. Chodankar et al. also used the Cre-lox system in C57/BL6 mice, driven off a truncated follicle stimulating hormone receptor promoter, to inactivate *Brca1 *in granulosa cells [21]. These conditional knockout mice developed cystic structures in both ovary and uterus, lined by cells of epithelial morphology, rather than granulosa cells, which had a normal *Brca1 *genotype. Two further transgenic models targeting the activin/Transforming Growth Factor-β (TGFβ) pathway in the ovary, one expressing a dominant-negative version of the TGF-β transcription factor *Smad2 *[22], and the other an inhibin α-subunit transgenic causing loss of activin activity [23], result in large serous cysts similar to those observed in this study, with ciliated and signet ring cyst cells reported. Both these transgenic mouse strains were developed on a CD-1 background, but cysts were not present in age-matched wildtype ovaries. It is notable that none of these models developed epithelial adenocarcinoma, reiterating that multiple mutational events are required for transformation of ovarian epithelial cells [24,25].

Low rates of BrdU incorporation were seen in all three epithelial compartments of the ovary, with the lowest rates observed in the rete ovarii and highest rates in cyst epithelia. This is consistent with previous work showing a higher rate of PCNA immunoreactivity in cysts compared with OSE [26] and very low rates of Ki-67 staining in the rete ovarii [27]. Increased rates of BrdU incorporation in cysts and the larger size of cysts in older animals, may suggest the cysts are actively expanding, but it is also possible cyst cells have higher rates of DNA repair [28,29].

The OSE from older incessantly ovulated ovaries had significantly higher rates of BrdU incorporation than OSE from breeder ovaries, killed at the same time and stage of the estrous cycle. This result contrasts with our previous findings of low PCNA staining in the OSE of ovaries from 8-month IO mice [26]. Given the higher number of PCNA-stained or BrdU-stained cells observed at the base of ovulation sites ([26] and Figure 4C), the difference between the IO and BR groups in the current study might be explained by differences in the number of ovulation sites per ovary in the two groups at the same age. The total ovulation number for each animal was estimated, rather than measured (see Materials and Methods, as well as reference [1]) so differences in ovulation rate between the two groups were theoretically possible. However mean litter size declined only minimally in the breeders with age. Furthermore, the available follicle pool and thus the ovulation rate might be expected to be lower in older IO mice compared with breeders and therefore a difference in the number of ovulations per ovary does not explain the result observed. The increased BrdU incorporation rate seen in the OSE of older IO animals may also correlate with a higher rate of DNA repair in OSE cells involved in re-epithelialization after many ovulations, although the kinetics of BrdU incorporation during DNA repair make this unlikely [28,29]. While the breeder females were not pregnant at time of death, they had been pregnant consistently since the age of 6-weeks and therefore would have been exposed to high levels of pregnancy hormones for a long period of time. Progesterone has been shown to inhibit OSE proliferation [30] and is thought to be protective against epithelial ovarian cancer [31,32]. The lower rates of BrdU incorporation seen *in vivo *in OSE from older breeder ovaries therefore supports the inhibitory effects of progesterone on OSE proliferation observed *in vitro*. Conversely, the increased BrdU incorporation observed in the OSE of OI ovaries may reflect differential gonadotropin stimulation of the OSE. Repeated ovulatory events, stimulated by more frequent luteinizing hormone (LH) pulses, might be expected to increase the rate of OSE cell proliferation *in vivo*, as has been demonstrated *in vitro *for murine, human and ovine OSE cells [33-37] and *in vivo *after gonadotropin-induced superovulation in mice [38,39].

In contrast, BrdU incorporation in cyst epithelial nuclei did not change significantly with either increasing age or ovulation number. This observation suggests either cell cycle regulation or rates of DNA repair differ between the OSE and cyst epithelial cells. The similarity of BrdU incorporation rate in cyst cells from the two groups suggests the protective role of progesterone does not extend to the cysts. However the cyst epithelium appears to be capable of responding to progesterone stimulation, since we (O. L. Tan and J. S Fleming, unpublished observations) and others [40-42] have shown strong expression of progesterone receptor protein in the nuclei of benign cystadenomas in both mouse and human. No correlation was demonstrated between ovarian cyst formation and circulating or cyst fluid steroid concentrations. Repeated ovulatory cycles, leading to a high total ovulation number, therefore do not cause significant changes in plasma androgen or estradiol-17β concentrations in mice.

E-cadherin protein expression was most pronounced in the rete ovarii of normal mouse ovaries, using our immunohistochemical conditions. This is in contrast with the results reported by Woolnough and colleagues [13], who found no evidence for E-cadherin expression in the human rete ovarii. E-cadherin is expressed by Mullerian duct-derived tissues such as oviduct and uterus, however [43]. The difference in expression pattern may be species-related, but direct comparison of E-cadherin immuno-staining in the mouse and human rete ovarii is required to verify this. Human OSE cells are more mesothelial than epithelial and express little or no E-cadherin, except occasionally in deep invaginations of OSE [9-11,44]. The peritoneal mesothelium also lacks E-cadherin [8]. Acquisition of E-cadherin immunoreactivity in cuboidal but not flat cells of human ovarian inclusion cysts, benign cystadenomas and primary low grade adenocarcinoma is well documented [9,12,45,46]. Loss of E-cadherin immunoreactivity in high grade tumors is significantly correlated to increased invasiveness, metastasis and poor prognosis [5,46]. In this study, apparent dilation of the rete ovarii was associated with a dramatic reduction in E-cadherin immunoreactivity in the flat cells lining cysts, similar to results reported in human ovaries [9]. Given the size of the mouse rete ovarii system relative to the size of the mouse ovary, similar dilation of the rete tubules in the much larger human ovary might not be noticed pathologically. The three cortical inclusion cysts showed variable E-cadherin immunoreactivity, with one showing little staining and the two papillary cysts demonstrating strong staining around cells. The pattern of loss of E-cadherin immunoreactivity as inclusion cysts form in mouse ovaries is consistent with previous observations in human cystadenomas [9,10,12,44]. However these experiments demonstrate that E-cadherin alone cannot be used to determine the cellular origin of the inclusion cysts in mice, since two papillary cortical inclusion cysts showed strong immuno-staining, despite not being connected to the ovarian hilus and therefore potentially to the rete ovarii. These data also suggest epithelial to mesenchymal transition is possible in the cortical cyst cells from mouse ovaries.

Extremely low levels of apoptosis were measured by either activated-caspase-3 immunohistochemistry or TUNEL analysis in any of the ovarian epithelial compartments and the few stained cells observed did not favor any particular age or treatment group. In mice, basal rates of apoptosis in the OSE have been reported at around 3% [47], but a recent study found virtually no apoptosis in the OSE, even after super-ovulation [38]. In our previous studies using scanning electron microscopy of the mouse ovary, incessant ovulation induced higher rates of invagination and apparent stratification of the OSE [1]. The low rates of apoptosis observed in the OSE in these experiments, in both BR and IO groups, combined with the higher rates of BrdU incorporation in the OSE of IO mice, suggest incessant ovulation affects cell proliferation, rather than apoptosis, in the OSE.

## Conclusion

The results of this age-matched study suggest longer periods of incessant ovulation do not lead to significant changes in inclusion cyst formation or steroidogenesis in CD-1 mice. Inclusion cysts developed with age, rather than total lifetime ovulation number. Incessant ovulation has direct effects on the OSE in this mouse strain, increasing cell proliferation and/or DNA damage repair, thus potentially increasing the chance of invagination and cortical cyst formation, but does not affect circulating steroid concentrations. The pattern of E-cadherin expression suggests there is heterogeneity in CD-1 mouse ovarian cyst structure, similar to that seen in human benign serous cystadenomas. We conclude that cortical and hilar cysts cannot be distinguished by morphology, cell proliferation rates or E-cadherin immuno-staining characteristics.

## Competing interests

The author(s) declare that they have no competing interests.

## Authors' contributions

HJM carried out the monitoring of estrous cycles, tissue collection, immunohistochemistry, participated in the analysis of ovarian histology and drafted parts of the manuscript. MJM carried out the steroid radioimmunoassays, monitoring of estrous cycles and tissue collection. CRB carried out the monitoring of estrous cycles and tissue collection and conducted the studies on bromodeoxyuridine incorporation and apoptosis, as well as drafting and editing parts of the manuscript. VL performed the statistical analysis. JSF conceived of the study, and participated in its design and coordination, supervised the laboratory work and wrote the manuscript. All authors read and approved the final manuscript.
